# The Role of PPAR Gamma in Systemic Sclerosis

**DOI:** 10.1155/2015/124624

**Published:** 2015-05-06

**Authors:** Andréa Tavares Dantas, Michelly Cristiny Pereira, Moacyr Jesus Barreto de Melo Rego, Laurindo Ferreira da Rocha, Ivan da Rocha Pitta, Cláudia Diniz Lopes Marques, Angela Luzia Branco Pinto Duarte, Maira Galdino da Rocha Pitta

**Affiliations:** ^1^Serviço de Reumatologia do Hospital das Clínicas da Universidade Federal de Pernambuco (HC-UFPE), 50670-901 Recife, PE, Brazil; ^2^Laboratório de Imunomodulação e Novas Abordagens Terapêuticas da Universidade Federal de Pernambuco (LINAT-UFPE), 50670-901 Recife, PE, Brazil; ^3^Laboratório de Planejamento e Síntese de Fármacos da Universidade Federal de Pernambuco (LPSF-UFPE), 50670-901 Recife, PE, Brazil

## Abstract

Fibrosis is recognized as an important feature of many chronic diseases, such as systemic sclerosis (SSc), an autoimmune disease of unknown etiology, characterized by immune dysregulation and vascular injury, followed by progressive fibrosis affecting the skin and multiple internal organs. SSc has a poor prognosis because no therapy has been shown to reverse or arrest the progression of fibrosis, representing a major unmet medical need. Recently, antifibrotic effects of PPAR*γ* ligands have been studied *in vitro* and *in vivo* and some theories have emerged leading to new insights. Aberrant PPAR*γ* function seems to be implicated in pathological fibrosis in the skin and lungs. This antifibrotic effect is mainly related to the inhibition of TGF-*β*/Smad signal transduction but other pathways can be involved. This review focused on recent studies that identified PPAR*γ* as an important novel pathway with critical roles in regulating connective tissue homeostasis, with emphasis on skin and lung fibrosis and its role on systemic sclerosis.

## 1. Introduction

Fibrosis is defined as an inappropriate repair by connective tissue characterized by excessive deposition of collagen and other extracellular matrix (ECM) components, promoting disruption of tissue homeostasis. It is recognized as an important feature of many chronic diseases, including myocardial infarction, glomerulosclerosis, idiopathic pulmonary fibrosis, liver cirrhosis, and systemic sclerosis (SSc) [1].

Fibroblasts are major effector cells in the development of fibrosis and an inappropriate fibroblast activation is the fundamental pathogenic alteration underlying pathologic fibrosis. A subgroup of resident fibroblasts, in response to transforming growth factor-*β* (TGF-*β*) stimulation, transdifferentiate into myofibroblasts expressing high levels of *α*-smooth muscle actin (*α*-SMA) with a significant functional role in pathologic fibrosis. The myofibroblasts show accelerated synthesis of extracellular matrix proteins, are resistant to apoptosis, and have contractile properties. Furthermore, bone-marrow-derived mesenchymal progenitors such as fibrocytes and monocytes might traffic to damaged tissue and undergo* in situ* differentiation into activated fibroblasts and myofibroblasts. Nonfibroblastic cell lineages (such as epithelial or endothelial cells or adipocytes) can also differentiate into fibroblasts and myofibroblasts through a process called epithelial-mesenchymal transition (EMT) [2–4].

Regulation of these cellular transitions, collagen gene expression, and ECM accumulation is tightly controlled. Various chemokines/cytokines can induce cell migration and proliferation, as well as stimulation of cell-cell adhesion and collagen production, which is associated with the pathogenesis of fibrosis. TGF-*β* is considered the main regulator of physiologic fibrogenesis and pathological fibrosis, and it has emerged as an important therapeutic target in fibrotic diseases [5, 6].

Intracellular TGF-*β* signaling is primarily mediated via the canonical Smad pathway. Binding of TGF-*β* to type 2 TGF-*β* receptor recruits type 1 TGF-*β* receptors (TGF-*β*RI), forming a heterotetrameric structure that phosphorylates Smad2 and Smad3, which then binds to Smad4. The resulting Smad complex then translocates to the nucleus and binds to the Smad binding elements (SBE) in the gene promoter in order to regulate the transcription of target genes [7]. Smads regulate transcription of target genes by interacting with other transcription factors and by recruiting transcriptional coactivators or corepressors, such as CREB (cAMP response element binding protein) binding protein (CBP)/p300 [8].

Although the Smad pathway is the central intracellular mediator of signals from the TGF-*β* receptors, recent evidence indicates that alternative non-Smad pathways exist. This also mediates TGF-*β* responses, involving protein kinases (MAP kinases p38 and JNK, focal adhesion kinase FAK, and TGF-*β* activated kinase TAK1), lipid kinases such as PI3 kinase and its downstream target Akt, the calcium-dependent phosphatase calcineurin, and the nonreceptor tyrosine kinase c-Abl [9, 10].

Besides TGF-*β*, multiple cytokines, growth factors, and chemokines regulate collagen production, ECM accumulation, and mesenchymal cell function and are also expressed abnormally in fibrotic diseases. These mediators, such as connective tissue growth factor (CTGF), platelet-derived growth factor (PDGF), interleukin (IL)-4, IL-6, IL-13, and IL-8, interact with TGF-*β* and directly contribute to the pathogenesis of fibrosis and might also represent potential targets for antifibrotic therapy [11].

Although the diagnosis and pathophysiology of most fibrosing diseases have been better characterized over the past few years, there remains no effective therapy for this group of diseases. Systemic sclerosis is an autoimmune disease of unknown etiology, characterized by immune dysregulation and vascular injury, followed by progressive fibrosis affecting the skin and multiple internal organs, mainly the lung. The disease has a poor prognosis because no therapy has been shown to reverse or arrest the progression of fibrosis, representing a major unmet medical need.

Therapies initially targeted to inflammation proved to be ineffective. Thus, studies have focused on modulation of profibrotic molecules, targeting myofibroblast differentiation, recruitment, and activity as a potential antifibrotic treatment. Hence, the transcription factor peroxisome proliferator-activated receptor gamma (PPAR*γ*) appears to participate in controlling fibrogenesis by inhibiting the TGF-*β* pathway. Aberrant PPAR*γ* function seems to be implicated in pathological fibrosis in the skin, lung, liver, heart, kidney, and pancreas. This review focused on recent studies that identified PPAR*γ* as an important novel pathway with critical roles in regulating connective tissue homeostasis, with emphasis on skin and lung fibrosis and its role in systemic sclerosis.

## 2. Role of PPAR***γ*** in Fibrosis Signaling

PPAR*γ* is a ligand-dependent nuclear receptor that belongs to the nuclear hormone receptor superfamily and regulates the expression of target genes. Some studies demonstrated the pivotal role of PPAR*γ* in glucose homeostasis, lipid metabolism, and cell growth regulation and, posteriorly, in inflammation, innate immunity, and regulation of connective tissue biology [12]. It is now recognized that PPAR*γ* modulates connective tissue synthesis and degradation, mesenchymal cell activation, transdifferentiation, and survival [13].

PPAR*γ* is activated by natural and pharmacological agents. Endogenous ligands include 15-deoxy−Δ12,14-prostaglandin J2 (15d-PGJ2), lysophosphatidic acid, and nitrolinoleic acid. PPAR*γ* can also be activated by synthetic ligands including the thiazolidinediones (TZD) as well as oleanic acid derivatives known as triterpenoids (2-cyano-3,12-dioxoolean-1,9-dien-28-oic-acid (CDDO)) [14]. The TZDs are highly potent PPAR*γ* agonists. They consist of rosiglitazone (RGZ), pioglitazone (PGZ), and troglitazone (TGZ) and were originally approved for the treatment of type 2 diabetes but their commercialization has been questioned in many countries mainly due to cardiovascular safety profile and increased risk of cancer and bone fractures [15–17].

In unstimulated cells, the PPAR*γ* receptors are located in the cytoplasm as heterodimers complexed to their repressors. After ligation with its agonist, PPAR*γ* heterodimerizes with retinoid X receptor (RXR) and coactivators such as p300 are recruited; this complex is translocated to the nucleus where it recognizes specific DNA sequence elements termed as peroxisome proliferator response element (PPRE) in promoters of target genes. PPARs regulate numerous genes through ligand-dependent transcriptional activation and repression [18, 19]. In the absence of ligands, the PPAR/RXR complex is bound to transcriptional corepressors and histone deacetylases, which prevents its binding to PPRE [13].

PPAR*γ* express two isoforms: PPAR*γ*1, present in macrophages, colonic epithelial cells, endothelial cells, and vascular smooth muscle cells, and PPAR*γ*2, mainly expressed in adipose tissue associated with the regulation of adipogenesis. The expression level of PPAR*γ* in a given cell or tissue determines the intensity and duration of the cellular response to endogenous or synthetic PPAR*γ* ligands [6, 19].

Recent studies have established that PPAR*γ* is a negative regulator of profibrotic signal-induced collagen synthesis and blunts fibrogenesis in a wide variety of organs. The antifibrotic effects of PPAR*γ* ligands were studied* in vitro* and* in vivo* and some theories have emerged leading to new insights. Indeed, it is possible that they act through a variety of distinct mechanisms according to different cell types or type of agonist (natural or synthetic) [13, 20–27].

An inverse relationship between fibrosis and PPAR*γ* expression/function was reported in multiple human fibrosing disorders as well as in animal models of fibrosis. Under physiologic conditions, PPAR*γ* shows a low level of constitutive activation, driven by natural ligands controlling fibrotic responses. Prolonged or recurrent fibrogenic stimulation decreases the expression of PPAR*γ*, inhibiting cellular responsiveness to natural endogenous PPAR*γ* ligands. In multiple organ-specific human fibrotic diseases, fibrosis is preceded by reduced tissue PPAR*γ* levels, suggesting a causal role for reduced PPAR*γ* expression or activity in the development or progression of fibrosis [13]. It is not clear in these conditions whether fibrosis is the cause of reduced PPAR*γ* or whether reduced PPAR*γ* causes fibrosis [6].

Some cytokines and chemokines are recognized as regulators of PPAR*γ* expression. Cytokines implicated in fibrosis generally suppress PPAR*γ* expression in mesenchymal effector cells. As an example, TGF-*β* seems to reduce PPAR*γ* expression in fibroblasts and hepatic stellate cells, although it stimulates PPAR*γ* expression in monocytes and macrophages [13]. Other inhibitors of PPAR*γ* expression include CTGF, IL-13, Wnt, leptin, lysophosphatidic acid (LPA), and hypoxia [28, 29].

**Figure 1:**
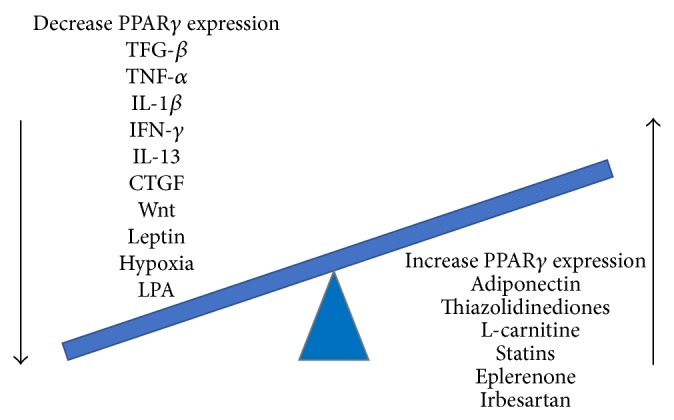
Effects of different molecules on PPAR*γ* expression.

On the contrary, adiponectin, which is regulated itself by PPAR*γ*, enhances the expression of PPAR*γ* in the liver and adipose tissue [30, 31]. Some molecules (L-carnitine, eplerenone, statins, and irbesartan) were studied as potential antifibrotic agents because of their effect on increasing PPAR*γ* expression [32–35] (Figure 1).

The molecular pathways underlying the antifibrotic effects of PPAR*γ* are not completely defined. One of the proposed mechanisms is the antagonistic relationship between PPAR*γ* and TGF-*β* signaling in fibrosis. As previously discussed, TGF-*β* promotes myofibroblasts differentiation from fibroblasts. In contrast, PPAR*γ* ligands induce adipogenic differentiation of skin fibroblasts [21]. TGF-*β* negatively regulates both the expression and function of PPAR*γ*, thereby desensitizing fibroblasts to PPAR*γ* ligands. On the other hand, PPAR*γ* ligands can directly disrupt TGF-*β* signal transduction and suppress TGF-*β* production [13, 21].

Activation of PPAR*γ* by either naturally occurring or synthetic ligands inhibits the induction of profibrotic responses induced by TGF-*β* in fibroblasts. While the effects of PPAR*γ* ligands (15d-PGJ2 and troglitazone) on collagen expression were only modest in unstimulated skin fibroblasts, these ligands significantly prevented collagen synthesis and expression in TGF-*β*-stimulated fibroblasts [21, 25–27]. PPAR*γ* agonists (troglitazone, 15d-PGJ2, and CDDO) also prevented *α*-SMA expression induced by TGF-*β* in skin fibroblasts [25, 27]. In hepatic stellate cells, skin fibroblasts, and aortic muscle cells, PPAR*γ* ligands suppressed CTGF expression induced by TGF-*β*1 [36, 37].

In normal fibroblasts, PPAR*γ* ligands can inhibit profibrotic signaling triggered by TGF-*β* and can interfere with downstream signal transduction. Blockage of the canonical Smad signaling pathway was demonstrated by some authors [26, 27, 38]. In hepatic stellate cells, PPAR*γ* ligands prevented Smad3 phosphorylation [38]. In contrast, in the TGF-*β*-mediated fibroblast activation, PPAR*γ* agonists did not prevent Smad2/3 phosphorylation or nuclear accumulation, but, instead, prevented recruitment of the coactivator p300 to the transcriptional complex [39]. In cultures of explanted normal fibroblasts, the PPAR*γ* agonist CDDO prevented fibrogenic responses induced by TGF-*β*. Such effects occurred via disruption of Smad-dependent transcription, but without preventing Smad2/3 activation, and were also associated with inhibition of Akt activation [27].

In contrast, in dermal fibroblasts, rosiglitazone treatment did not attenuate expression of phosphorylated Smad2, suggesting that PPAR*γ* ligands can abrogate TGF-*β*-induced responses independent of Smad activation [21, 25, 40]. For example, rosiglitazone reduced the induction of Egr-1, an early immediate transcription factor of TGF-*β* signaling [41]. Studies also implicate upregulation of the tumor suppressor phosphatase and tensin homolog (PTEN) as responsible for the inhibition of profibrotic effector functions by PPAR*γ*.* In vitro* studies showed that PTEN inhibits fibroblast-myofibroblast differentiation and expression of *α*-SMA and collagen in human and mouse lung fibroblasts [42]. Accordingly, 15d-PGJ2 inhibited transcription of the TGF-*β*1 gene via PTEN upregulation in mouse fibroblasts [43].

In mesangial cells, PPAR*γ* ligands (15d-PGJ2, troglitazone, and ciglitazone) stimulated the expression of hepatocyte growth factor (HGF), an endogenous antifibrotic agent. HGF induces the Smad transcriptional corepressor TG-interacting factor (TGIF) thus mediating autocrine suppression of TGF-*β*-induced fibrogenic responses [44, 45].

**Figure 2:**
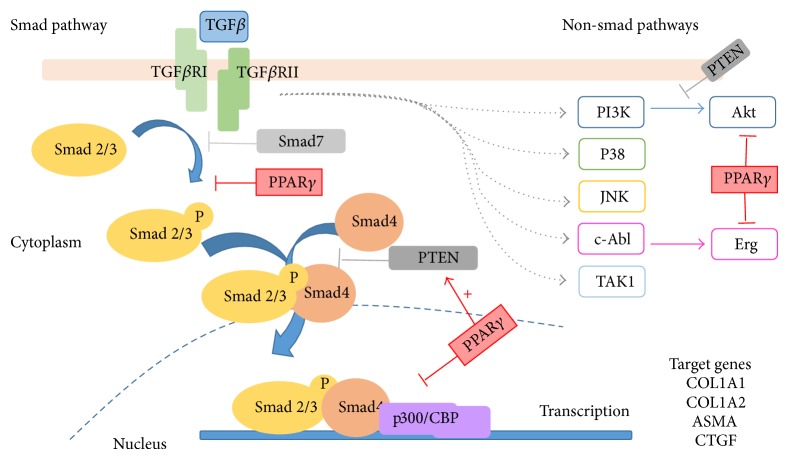
Smad and non-Smad signaling TGF-*β* pathways and potential effects of PPAR*γ* ligands. Binding of TGF-*β* to type 2 TGF-*β* receptor (TGF-*β*RII) recruits type 1 TGF-*β* receptors (TGF-*β*RI), forming a heterotetrameric structure that phosphorylates Smad2 and Smad3, which then binds to Smad4. Smad complex then translocates to the nucleus, where it interacts with various transcription factors to regulate the transcription of target genes (COL1A1, COL1A2, ASMA, CTGF). After TGF-*β* binding, TGF-*β*RII recruits a TGF-*β*RI and activates it by phosphorylation. Nonclassic pathways are also illustrated. PPAR*γ* ligands can block TGF-*β* signaling by blocking Smad and non-Smad pathways.

Contrary to the mentioned findings, some studies suggested that antifibrotic effects of PPAR*γ* ligands could not be related to PPAR*γ* activation [46–49]. Ferguson et al. demonstrated that CDDO inhibited *α*-SMA expression by a PPAR*γ*-independent mechanism, promoting dysregulation of acetylation of the TGF-*β* gene transcription coactivator CBP/p300 [48]. Similarly, Kulkarni et al. showed that PPAR*γ* ligands inhibited TGF*β*-induced Akt phosphorylation and this effect was not restored by PPAR*γ* antagonist [49].  Figure 2 illustrates some effects of PPAR*γ* in TGF*β* signaling pathway.

These data argue that PPAR*γ* agonists have a role in limiting fibrosis, in addition to their already known anti-inflammatory and immunomodulatory effects. Tables 1 and 2 summarize* in vitro* and* in vivo* studies of antifibrotic effects of PPAR*γ* agonists. This antifibrotic effect is mainly related to the inhibition of TGF-*β*/Smad signal transduction but other pathways may be involved. This knowledge has stimulated the development of further studies examining PPAR*γ* role in fibrotic diseases and the potential therapeutic use of their ligands.

## 3. PPAR***γ*** and Lung Fibrosis

Lung fibrosis occurs in a wide variety of illnesses, including systemic disorders, as systemic sclerosis, as well as primary lung disease, such as idiopathic interstitial pneumonia (IIP). Fibrotic remodeling of lung tissue is also an important feature of other lung diseases, including sarcoidosis, asthma, and chronic obstructive pulmonary disease. In general, it is characterized by inflammatory cell infiltration and alveolar epithelial cell injury with failure of alveolar reepithelialization, followed by recruitment and persistence of fibroblasts that differentiate into myofibroblasts. The excessive collagen and extracellular matrix production results in distortion of the lung architecture and consequently decreased gas exchange and reduced pulmonary compliance [50, 51].

Many types of lung cells express PPAR*γ*, including fibroblasts, T lymphocytes, ciliated airway epithelial cells, alveolar type II pneumocytes, alveolar macrophages, and airway smooth muscle cells [52]. Reduced PPAR*γ* expression was demonstrated in lung fibroblasts from patients with SSc [22, 53] and in alveolar macrophages of patients with sarcoidosis [54] and pulmonary alveolar proteinosis [55], suggesting that insufficient PPAR*γ* activity may contribute to ongoing dysregulated inflammation and fibrosis.

PPAR*γ* ligands have negative regulatory effects on human lung fibroblasts, by inhibiting proliferation and migration of healthy or IIP fibroblasts and by inhibiting proliferative responses of undifferentiated fibroblasts and myofibroblasts to mitogenic growth factors, as PDGF [47, 56]. Furthermore, PPAR*γ* agonists inhibited the human lung fibroblast transdifferentiation mediated by TGF-*β* to the myofibroblast phenotype [40, 47, 48, 56] and significantly reduced expression of fibronectin [48] and type I collagen TGF-*β*-stimulated [40, 47].

TGF-*β* is a potent stimulus for induction of pulmonary fibrosis* in vivo* [57]. Wei et al. demonstrated that normal lung fibroblasts stimulated with TGF-*β* showed a decrease in PPAR*γ* expression [22]. In another experiment, primary lung fibroblasts showed a small and not significant increase, followed by an expressive downregulation of PPAR*γ* expression after exposure to TGF-*β*, beginning after an hour and persisting for at least 48 hours. This effect was reduced in Smad3-deficient lung fibroblasts, suggesting that TGF-*β*1 modulates PPAR*γ* expression, in part, via Smad3 signaling. Additionally, the inhibition of transcriptional ability of PPAR*γ* by TGF-*β*1 was overcome by overexpression of PPAR*γ* [24].

Other mechanisms are also proposed to explain PPAR*γ* agonists action. Activation of ERK-MAPK pathway by TGF-*β* plays an important role in fibrosis by regulating myofibroblast transdifferentiation, cell proliferation, and survival, as well as ECM synthesis [58]. In lung fibroblasts, RGZ showed an antifibrotic effect by decreasing ERK phosphorylation induced by PDGF and TGF-*β*1 [56].

Recent studies provide evidence that alveolar epithelial cells (AEC) can undergo a TGF-*β*1-induced epithelial-mesenchymal transition (EMT), acquiring a fibroblast-like phenotype and possibly contributing to lung fibrosis. Phenotypic markers associated with EMT include the diminished expression of E-cadherin, a cell anchoring protein expressed specifically by epithelial cells, and an elevated expression of N-cadherin, normally present at relatively high levels in fibroblasts. Tan et al. demonstrated that RGZ and CGZ inhibited the elevation of markers of profibrotic phenotype (N-cadherin, CTGF, and collagen I) in TGF-*β*1-stimulated A549 cells, a model of AEC type II [59].

Studies with animal models found that the PPAR*γ* agonists troglitazone [47], pioglitazone [60], and rosiglitazone [61, 62] were able to inhibit lung fibrosis bleomycin induced. This inhibition was observed either before or even after bleomycin administration. The initial period of postinflammatory fibrosis could correspond to the period in which patients are likely to present symptoms [47, 60]. More recently, using microcomputed tomography to evaluate radiological changes in the murine model of lung fibrosis, two authors demonstrated that the treatment of bleomycin-instilled mice with RGZ prevented the development of [63] or improved [64] typical features of lung fibrosis, like ground glass opacity and consolidation.

**Figure 3:**
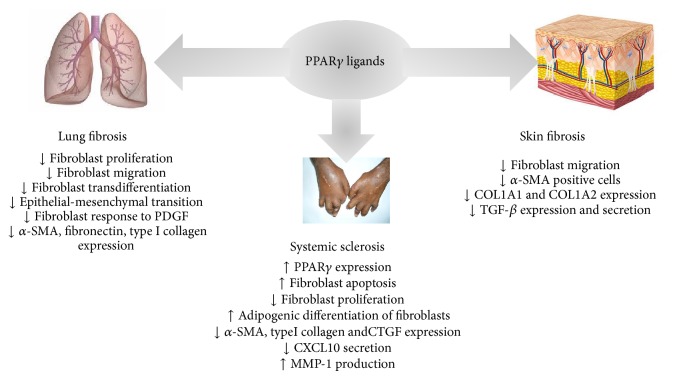
Effects of PPAR*γ* ligands in fibrotic diseases.

In conclusion, there are some evidences that PPAR*γ* agonists have antifibrotic effects on human lung fibroblasts, as demonstrated by the attenuation of bleomycin-induced lung injury and downregulation of TGF-*β*1-mediated collagen deposition in fibrotic lung tissues (Figure 3).

## 4. PPAR***γ*** and Skin Fibrosis

Wound repair is a very complex and dynamic process, involving the interactions of multiple cell types and growth factors, cytokines, and soluble mediators. Normal cutaneous tissue repair involves an initial inflammatory phase, characterized by migration of inflammatory cells to the injured site, followed by a fibroproliferative phase, with synthesis and deposition of granulation tissue and neovascularization. Finally, the resolution phase is characterized by replacement of damaged and granulation tissue by newly synthesized fibrous matrix protein collagens. In response to tissue injury, myofibroblasts repopulate the wound and synthesize and remodel new ECM. Dysregulation of this process could result in chronic wounds or fibrosis [65, 66].

It is suggested that PPAR*γ* may in part be responsible for initiating endogenous mechanisms of wound repair and the activation of PPAR*γ* by its natural ligands controls fibrotic responses. Normal dermal fibroblasts constitutively express low levels of PPAR*γ*, distributed in both nucleus and cytoplasm [21, 25, 67]. Kapoor et al. demonstrated that PPAR*γ* is upregulated during the resolution phase of normal wound healing [68]. Besides, PGJ2 physiologically increases and there is an upregulation of PPAR*γ* expression, leading to blocking of fibroblast activation and collagen neosynthesis [6, 68]. Migration of dermal fibroblasts plays a critical role in both normal wound healing and pathological fibrogenesis. Treatment with rosiglitazone abrogated stimulation of fibroblast migration and wound closure elicited by TGF-*β* [41].

In TGF-*β*-stimulated dermal fibroblasts, there was a significant time-dependent decrease in PPAR*γ* expression and a similar inhibition of matrix metalloproteinase- (MMP-) 1 and Smad3. At the same time, there was an increase in the expression of fibrosis-related genes such as ASMA, SERPINE1, CTGF, and COMP1 [22]. In addition, it was demonstrated* in vivo *a decline in cutaneous PPAR*γ* expression in a mouse model of bleomycin-induced skin fibrosis [41].

Skin fibrosis associated with progressive loss of PPAR*γ* expression seems to be prevented or reduced by the administration of PPAR*γ* ligands. In the model of bleomycin-induced skin fibrosis, treatment with PPAR*γ* ligands (RGZ, CDDO) prevented the development of skin fibrosis and also reduced established fibrosis [21, 27]. Gonzalez et al., using ajulemic acid (AjA), a nonpsychoactive synthetic analogue of tetrahydrocannabinol that can bind to PPAR*γ*, also demonstrated prevention of experimental bleomycin-induced dermal fibrosis and interruption of further progression of established fibrosis, but did not alter preexisting ECM accumulation [69].

In human dermal fibroblasts troglitazone reduced TGF-*β*1 secretion [67] and administration of rosiglitazone substantially prevented the upregulation of TGF-*β*1 [21]. PPAR*γ* agonists also attenuated the upregulation of the fibrogenic genes COL1A1 and COL1A2 and reduced the number of *α*-SMA-positive fibroblastic cells [21]. An interesting finding was that the overexpression of PPAR*γ* resulted in complete suppression of COL1A2 gene transcription stimulated by TGF-*β*, even in the absence of an activation ligand, and caused a sensitization of fibroblasts to the ligands. These observations suggest that the relatively low level of PPAR*γ* expression in normal fibroblasts may be a limiting factor for negative regulation of TGF-*β* responses [25].

Ghosh et al. found that basal type I collagen gene expression was markedly elevated in mouse embryonic fibroblasts (MEFs) lacking PPAR*γ*. The agonist 15d-PGJ2 failed to suppress the elevated levels of collagen in MEFs. At the same way, activity of the COL1A2 promoter was markedly elevated in PPAR*γ* null MEFs and reconstitution of these cells with ectopic PPAR*γ* resulted in downregulation of COL1A2 promoter activity. PPAR*γ* null MEFs displayed elevated expression of type I TGF-*β* receptor (T*β*RI) and produced more TGF-*β*1. Furthermore, PPAR*γ* null MEFs showed Smad2/3 phosphorylation, with nuclear accumulation, even in the absence of stimulation by exogenous TGF-*β*. These results indicate that absence of PPAR*γ* in MEFs is associated with constitutive upregulation of collagen gene expression and Smad activation, at least in part, due to autocrine TGF-*β* stimulation [70].

In animal models, PPAR*γ* null skin fibroblasts showed an enhanced responsiveness to tissue injury, as shown by increased rate of dermal wound closure, concomitant with increased collagen deposition, greater expression of *α*-SMA, CTGF, and proliferating cell nuclear antigen (PCNA), a marker of cell proliferation. They also showed elevated phosphorylation of Smad3, Akt, and ERK. Conversely, loss of PPAR*γ* expression by itself was not sufficient to promote skin fibrosis, since PPAR*γ*-deficient skin did not show significant alterations in skin thickness or matrix accumulation [71].

In line with these findings, Kapoor et al., using bleomycin-induced skin fibrosis in PPAR*γ* knockout mice, showed enhanced susceptibility to skin fibrosis as demonstrated by enhanced dermal thickness, higher scores for collagen content, and greater expression of *α*-SMA. PPAR*γ*-deficient mice also showed elevated Smad3 phosphorylation, indicating a potentiation of the profibrotic TGF*β*1/Smad signaling pathway in the absence of PPAR*γ*. TGF-*β*1-stimulated dermal fibroblasts isolated from PPAR*γ*-KO mice had an increase in expression of *α*-SMA and type I collagen [72]. Taken together, these findings suggest that PPAR*γ* normally suppresses fibrogenesis* in vivo* and that loss of PPAR*γ* expression in skin results in elevated profibrotic signaling [72, 73].

These data indicate that PPAR*γ* plays an important role in suppressing the skin fibrogenic response by antagonizing TGF-*β* signaling in physiological conditions and highlight the potential ability of PPAR*γ* agonists to inhibit abnormal synthesis and tissue accumulation of collagen in fibrotic diseases (Figure 3).

## 5. PPAR***γ*** and Systemic Sclerosis

Systemic sclerosis is a clinically heterogeneous disease, known as the most severe connective tissue disorder, and associated with a high mortality risk. Patients with SSc may exhibit proliferative small artery and obliterative microvascular disease. There is also inflammation and fibrosis affecting the skin, oesophagus, respiratory tract, and other target organs. Loss of cutaneous elasticity and accompanying tightness followed by thickening and hardening of the skin (sclerosis) is almost always present and it has an important impact on quality of life. Skin involvement is a marker of disease activity and presents correlation with disease prognosis [74]. Pulmonary involvement is also common in patients with SSc and most often comprises fibrosis or interstitial lung disease and pulmonary vascular disease leading to pulmonary arterial hypertension (PAH). Currently, pulmonary manifestations are the leading cause of disease-related morbidity and mortality in patients with SSc [75].

The pathological events in SSc are complex and include impaired communication between endothelial cells, epithelial cells, and fibroblasts; lymphocyte activation; autoantibody production; inflammation; connective tissue fibrosis. These events result in an accumulation of constituents of the ECM, which replaces the normal tissue architecture, thus culminating in organ failure [76]. Scleroderma fibroblasts display an activated phenotype characterized by overproduction of collagen, secretion of profibrotic cytokines and chemokines, and expression of cell-surface integrin adhesion molecules and receptors for TGF-*β*, PDGF, and CCL2. Furthermore, SSc fibroblasts show increased expression of *α*-SMA and resistance to apoptosis [77, 78].

Reduced expression of PPAR*γ* mRNA and protein was demonstrated in SSc skin biopsies, as well as in explanted skin fibroblasts [22, 69, 70, 79]. Although the cause underlying the PPAR*γ* deficit in SSc and other fibrosing conditions is not yet known, multiple factors implicated in the pathogenesis of fibrosis, such as TGF-*β*, CTGF, and IL-13, potently inhibit PPAR*γ* expression and function [3].

PPAR*γ* expression shows an inverse relationship with enhanced TGF-*β* signaling in SSc lesional tissue. Microarray-based expression profiling of SSc skin biopsies showed an inverse correlation between PPAR*γ* mRNA and levels of plasminogen activator inhibitor-1 (PAI-1), a TGF-*β*-regulated gene and marker of TGF-*β* activity [22].

Although fibroblasts from lesional SSc skin show reduced PPAR*γ* expression, treatment with PPAR*γ* ligands was able to increase the levels of the endogenous PPAR*γ* ligand 15d-PGJ2 and the PPAR*γ* expression [69, 79]. Furthermore, rosiglitazone attenuated the activated phenotype of scleroderma fibroblasts, by suppressing *α*-SMA, type I collagen, and CTGF protein expression and by reducing the ability of these fibroblasts to contract collagen matrix [79]. Other nonthiazolidinic PPAR*γ* ligands, AjA and CDDO, also reduced collagen neosynthesis by scleroderma fibroblasts* in vitro*, an action that was reversed completely by cotreatment with a selective PPAR*γ* antagonist [27, 69].

The expression of PPAR*γ* is also reduced in lung fibroblasts from SSc patients [22, 53]. Treatment with RGZ resulted in significantly increased levels of PPAR*γ* in SSc but not in normal lung fibroblasts. In addition, RGZ increased the production of MMP-1 and inhibited collagen type I, CTGF, and *α*-SMA expression [53]. Besides, RGZ or PGZ significantly reduced cell proliferation and viability and increased apoptosis in SSc fibroblasts, whereas they did not present a significant influence on healthy fibroblasts [80].

As mentioned above, the role of myofibroblasts as the principal mesenchymal cell responsible for the formation of fibrotic tissue is already well established in SSc and other fibrotic diseases. However, the origin of myofibroblasts is not completely understood. Recently, it was suggested that myofibroblasts in fibrotic skin could originate from adiponectin-positive intradermal progenitors via adipocyte-myofibroblast transition [81]. In line with this, development of dermal fibrosis is accompanied by progressive atrophy of the subcutaneous adipose layer and fibrous tissue replacement. An interesting finding is that PPAR*γ* ligands induced adipogenic differentiation of mature dermal fibroblasts as well as preadipocytes, and this process was reversed by TGF-*β* [21].

SSc patients showed reduced serum levels and skin expression of adiponectin. An inverse correlation between serum adiponectin levels and skin fibrosis was also observed in these patients [23, 82, 83]. Adiponectin is a direct transcriptional target of PPAR*γ*, whose levels directly reflect PPAR*γ* activity, and it could mediate the antifibrotic effects of PPAR*γ* [84, 85]. It was demonstrated that this adipokine suppressed the expression of type I collagen and *α*-SMA in normal and scleroderma fibroblasts and abrogated the stimulation of these responses elicited by TGF-*β* [85]. Thus, adiponectin levels might be a potential biomarker of the level of PPAR*γ* expression and progression of fibrosis.

Rosiglitazone attenuated the CXCL10/IP-10 secretion in explanted SSc fibroblasts, suggesting other potential effects of PPAR*γ* ligands in SSc apart from antifibrotic action [86]. CXCL10 has been implicated in SSc pathogenesis since increased serum levels and epidermis expression were demonstrated in SSc patients [87] in addition to an association with more severe clinical phenotype [88].

These studies demonstrated that PPAR*γ* expression and activity are reduced in SSc. This impaired PPAR*γ* expression resulting from its suppression by TGF-*β* and related cytokines might contribute to unregulated fibroblast activation and persistent fibrogenesis and represent an important advance in understanding the pathophysiology of SSc. Therefore, more studies are needed to evaluate the therapeutic potential of PPAR*γ* ligands in SSc (Figure 3).

## 6. Conclusion

Fibrosis is a major medical problem, which can lead to progressive dysfunction of many organs and eventually the death of patients. Many aspects of its molecular mechanisms are still unclear. Currently, no effective antifibrotic treatment is available. There are many studies suggesting a key physiologic function of PPAR*γ* signaling as an endogenous mechanism to prevent excessive fibrogenesis following injury. PPAR*γ* is a negative regulator of profibrotic signal-induced collagen synthesis and reduces fibrogenesis in a wide variety of organs in experimental animal models of fibrosis. Activation of cellular PPAR*γ* receptors using either synthetic or natural PPAR*γ* ligands blocks the induction of profibrotic responses. Experimental studies in systemic sclerosis demonstrated an impaired PPAR*γ* expression and function, supporting a potential pathogenic role of PPAR*γ* in this disease. Thus the use of synthetic agonists to induce the activation of PPAR*γ* signaling or to enhance defective PPAR*γ* tissue expression might be investigated as novel therapeutical approaches to the treatment of fibrosis.

## Figures and Tables

**Table 1:** *In vitro* studies of antifibrotic effects of PPAR*γ* agonists.

Cell type	PPARg Ligand	Effects	References
Healthy human lung fibroblast	15d-PGJ2, CGZ and RGZ	↓ TGF-*β*-induced myofibroblast differentiation ↓ TGF-*β*-induced type I collagen protein production	[40]

Normal lung fibroblasts and fibroblasts isolated from patients with IIP human fetal lung fibroblast (IMR-90) cells	CGZ and TGZ	↓ Proliferation of human lung fibroblasts ↓ Proliferative responses of undifferentiated Fibroblasts and myofibroblasts to PDGF Inhibited TGF-*β*1-induced myofibroblast differentiation	[47]

MRC-5 cells derived from human lung fibroblasts	PGZ	↓ TGF*β*-mediated increase in procollagen I and CTGF expression	[60]
	RGZ	↓ Lung fibroblast migration and proliferation ↓ Myofibroblast transdifferentiation	[56]

Normal human lung fibroblast cell	15 d-PGJ2, RGZ and CDDO	↓ TGF-*β*–stimulated differentiation of fibroblasts to myofibroblasts ↓ TGF-*β*–induced fibronectin expression	[48]

A549 human alveolar cell line	RGZ and CGZ	↓ Profibrotic changes (elevation of N-cadherin, CTGF and collagen I) in alveolar epithelial cells	[59]

Primary lung human fibroblasts Lung fibroblasts from IIP patients	CDDO and 15d-PGJ2	↓ TGF*β*-induced phosphorylation of Akt ↓ myofibroblast differentiation	[49]

SSc lung fibroblasts	RGZ	↑ MMP-1 expression ↓ Collagen type I expression in white patients ↓ CTGF and *α*-SMA expression	[53]

Primary cultures of human dermal fibroblasts	15d-PGJ2 and TGZ	↑ PPAR*γ* nuclear levels in skin fibroblasts ↓ type I collagen synthesis and expression by TGF*β*-stimulated fibroblasts ↓ *α*-SMA expression by TGF*β*-stimulated fibroblasts	[25]
	TGZ	↓ TGF*β*1, type I collagen and fibronectin expression and secretion	[67]

Human foreskin fibroblasts	15d-PGJ2 and TGZ	↓ Collagen synthesis and of COL1A2 promoter activity induced by TGF-*β* ↓ Smad3-dependent transcriptional responses without blocking Smad activation ↓ TGF*β*-induced interaction of p300 with Smad3 ↓ Recruitment of p300 to the DNA-bound transcriptional complex	[39]

Healthy and scleroderma fibroblasts	RGZ	↓ *α*-SMA, type I collagen and CTGF protein expression in dcSSc fibroblasts ↑ PPAR*γ* expression	[79]
	Ajulemic acid	↓ Supernatant levels of procollagen type I propeptide and TGFb	[69]

Human scleroderma fibroblasts	RGZ	Reduced CXCL10 secretion induced by IFN*γ* e TNF*α*	[86]
	PGZ and RGZ	↓ Cell proliferation and cell viability Increased apoptosis	[80]
	CDDO	↓ Cellular and secreted type I collagen levels ↓ COL1A1 and COL1A2 mRNA expression	[27]

Explanted normal human skin Fibroblasts	CDDO	↓ COL1A2 and aSMA expression induced by TGFb	[27]

Organotypic human skin raft model (epidermal keratinocytes and dermal fibroblasts)	CDDO	↓ COL1A1, COL1A2, and *α*-SMA expression in fibroblasts	[27]

Human A540 epithelial cells	CDDO	↓ TGF-*β*-induced epithelial–mesenchymal transition	[27]

CGZ = ciglitazone, RGZ = rosiglitazone, TGF-*β* = transforming growth factor-*β*, TGZ = troglitazone, IIP = idiopathic interstitial pneumonia, PDGF = platelet-derived growth factor, PGZ = pioglitazone, CTGF = connective tissue growth factor, CDDO = 2-cyano-3,12-dioxoolean-1,9-dien-28-oic-acid, SSc = systemic sclerosis, MMP-1 = matrix metalloproteinase-1, dSSc = diffuse systemic sclerosis.

**Table 2:** *In vivo* studies of antifibrotic effects of PPAR*γ* agonists.

Animal model	PPAR*γ* ligand	Effects	Reference
Bleomycin-induced model of lung fibrosis	15d-PGJ2 and RGZ	↓ Histological evidence of lung fibrosis	[61]
	TGZ	↓ Hydroxyproline and collagen deposition in lung tissue Ameliorated histopathological changes	[47]
	PGZ	↓ Hydroxyproline content in lung tissue Ameliorated histopathological changes	[60]
	RGZ	Prevented onset of fibrotic radiological changes	[63]
	RGZ	↓ Hydroxyproline content in lung tissue ↓ Lung TGF-*β*1 concentration Ameliorated histopathological changes	[62]

Bleomycin-induced model of skin fibrosis	RGZ	Attenuated severity of dermal fibrosis and local collagen deposition ↓ Tissue accumulation of myofibroblasts ↓ Levels of TGF-*β* levels in lesional skin	[41]
	Ajulemic acid	Prevented development of skin fibrosis ↓ Skin thickness dermal ↓ Hydroxyproline content ↓ Myofibroblasts number	[69]
	CDDO	↓ Collagen deposition and dermal thickness ↓ *α*-SMA and TGF*β*1 expression	[27]

Constitutively active TGF*β* receptor type I mouse model (AdTGFbRI)	Ajulemic acid	Prevented development of skin fibrosis ↓ Skin thickness dermal ↓ Hydroxyproline content ↓ Myofibroblasts number	[69]

RGZ = rosiglitazone, TGZ = troglitazone, PGZ = pioglitazone, CDDO = 2-cyano-3,12-dioxoolean-1,9-dien-28-oic-acid, TGF-*β* = transforming growth factor-*β*, *α*-SMA = *α*-smooth muscle actin.
